# Adsorption Properties of Doxorubicin Hydrochloride onto Graphene Oxide: Equilibrium, Kinetic and Thermodynamic Studies

**DOI:** 10.3390/ma6052026

**Published:** 2013-05-15

**Authors:** Shaoling Wu, Xindong Zhao, Yanhui Li, Qiuju Du, Jiankun Sun, Yonghao Wang, Xin Wang, Yanzhi Xia, Zonghua Wang, Linhua Xia

**Affiliations:** 1 Department of Hematology, Shandong Provincial Hospital, Shandong University, Jinan, Shandong 250021, China; E-Mail: qdwushaoling@163.com; 2 Department of Hematology, the Affiliated Hospital of medical College, 16 Jiangsu Road, Qingdao, Shandong 266003, China; E-Mails: zhaoxindong@yahoo.com.cn (X.Z.); duqiuju999@gmail.com (Q.D.); kun3812@163.com (J.S.); wangyonghao1988@sina.com (Y.W.); qdxyzh@163.com (Y.X.); wang_zonghua@yahoo.com.cn (Z.W.); autumnred@hotmail.com (L.X.); 3 Laboratory of Fiber Materials and Modern Textile, The Growing Base for State Key Laboratory, Qingdao University, 308 Ningxia Road, Qingdao, Shandong 266003, China

**Keywords:** doxorubicin hydrochloride, graphene oxide, adsorption, isotherm, kinetic

## Abstract

Doxorubicin hydrochloride (DOX) is an effective anticancer agent for leukemia chemotherapy, although its clinical use has been limited because of its side effects such as cardiotoxicity, alopecia, vomiting, and leucopenia. Attention has been focussed on developing new drug carriers with high adsorption capacity and rapid adsorption rate in order to minimize the side effects of DOX. Graphene oxide (GO), a new type of nanomaterial in the carbon family, was prepared by Hummers method and used as adsorbent for DOX from aqueous solution. The physico-chemical properties of GO were characterized by transmission electron microscope (TEM), Fourier transform infrared spectroscopy (FTIR), zeta potential, and element analysis. The adsorption properties of DOX on GO were studied as a function of contact time, adsorbent dosage, temperature and pH value. The results showed that GO had a maximum adsorption capacity of 1428.57 mg/g and the adsorption isotherm data fitted the Langmuir model. The kinetics of adsorption fits a pseudo-second-order model. The thermodynamic studies indicate that the adsorption of DOX on GO is spontaneous and endothermic in nature.

## 1. Introduction

Doxorubicin hydrochloride (DOX), an anthracycline ring antibiotic, is a highly effective anti-neoplastic agent used in leukemia chemotherapy. However, the severe toxic side-effects such as cardiotoxicity, alopecia, vomiting, leucopenia, and stomatitis have hampered the successful use of DOX. To reduce the undesired effects without reducing drug potency, DOX is usually encapsulated into drug delivery vehicles that have the ability to protect the molecule of interest and selectively target specific compartments without adversely affecting the surrounding tissues [1]. Currently, commonly used drug delivery vehicles include chitosan [2], liposomes [3], polymers [4], silica [5], nanoparticles [6], and carbon nanotubes [7]. Among them, carbon nanotubes have been considered as the most advanced nanovehicles for the highly efficient delivery of drugs and biomolecules owing to their large specific surface area, high chemical stability, as well as their unique optical and electrical properties. Graphene oxide (GO), a new emergent nanomaterial with one-atom-thick two-dimensional individual sheet structure composed of sp^2^-hybridized carbon, has attracted increasing interest in the field of biological detection, drug delivery, and cancer therapies [8].

The surface of GO prepared through the chemical oxidation of graphite has covalently attached oxygen-containing groups such as hydroxyl, carboxyl, and epoxy groups, which endow a capability for further surface chemical modification via noncovalent [9] and covalent functionalization methods [10]. The functionalized GO has low cytotoxicity and excellent solution solubility and biocompatibility. For instance, GO functionalized by polyethylene glycol readily complexes with a water insoluble aromatic molecule SN38 (a camptothecin analogue) via noncovalent van der Waals interaction. The obtained material not only water soluble, but also has a high toxicity which exceeds that of irinotecan (a commonly used prodrug for colon cancer treatment) by 2–3 orders of magnitude [11]. Studies *in vitro* showed that chemically derived GO hardly entered the A549 cell and has no obvious cytotoxicity at low concentration [12]. In an *in vivo* experiment dextran conjugated GO accumulated in the reticuloendothelial system including liver and spleen after intravenous injection, but did not cause noticeable short-term toxicity to the treated mouse [8].

The unique structure and exceptional physico-chemical properties of GO make it suitable for adsorbing heavy metal ions such as Pb^2+^, Cd^2+^, and Cu^2+^ [13,14,15], organics such as methylene blue [16], naphthalene [17] and 1-naphthol [18] as well as drug molecules such as tetracycline [19] and sulfonamide antibiotics [20]. However, studies on using GO as drug nanovectors have only recently begun. There have only been several pilot researchers who have reported using GO as drug carriers [11,12]. Very little is known about the interaction mechanism between DOX and GO.

In this paper, the adsorption properties of DOX onto GO were investigated systematically. The influencing factors such as contact time, dosage, pH value and temperature on the adsorption properties were studied. Analysis of the adsorption of DOX onto GO will benefit from understanding the interaction mechanism between the adsorbate and the adsorbent and serves as the basis for the establishment of nanoscale drug delivery systems.

## 2. Results and Discussion

### 2.1. Characterization of GO and DOX/GO 

**Figure 1:**
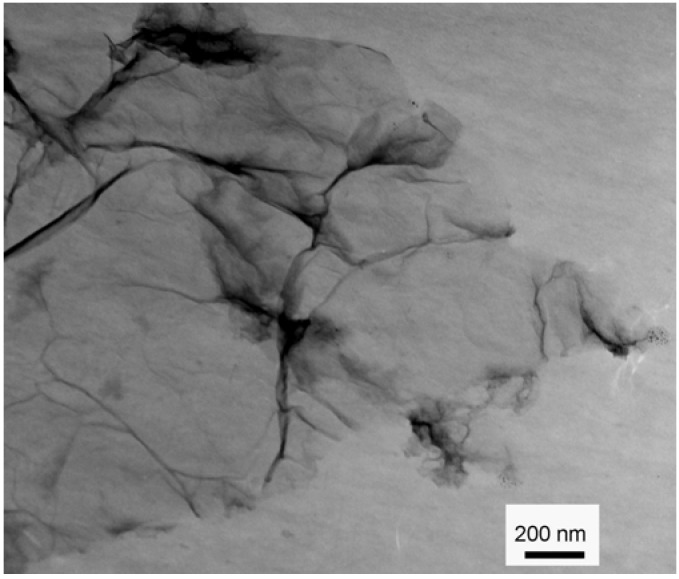
Transmission electron microscope (TEM) image of graphene oxide (GO).

The morphological structure of GO was characterized by transmission electron microscope (TEM) and shown in Figure 1. It is clear that GO is basically transparent. However, the elastic corrugations and the scrolled or folded edges often result in different brightness on the surface of the GO [21]. Elemental analysis showed the composition of GO as C, 45.17%; N, 0.78%; H, 3.15%; O, 50.90%. N_2_ adsorption-desorption experiments show that GO has a surface area of 32 m^2^/g, pore volume of 0.11 cm^3^/g, and average pore width of 17.3 nm, which is similar to the reported values [22]. The results indicate that GO has a low surface area and is a mesoporous material. The mesoporous structure and low surface area of GO may be due to the agglomerations of GO sheets during the drying treatment because of the unavoidable van der Waals force between each single sheet of GO.

**Figure 2:**
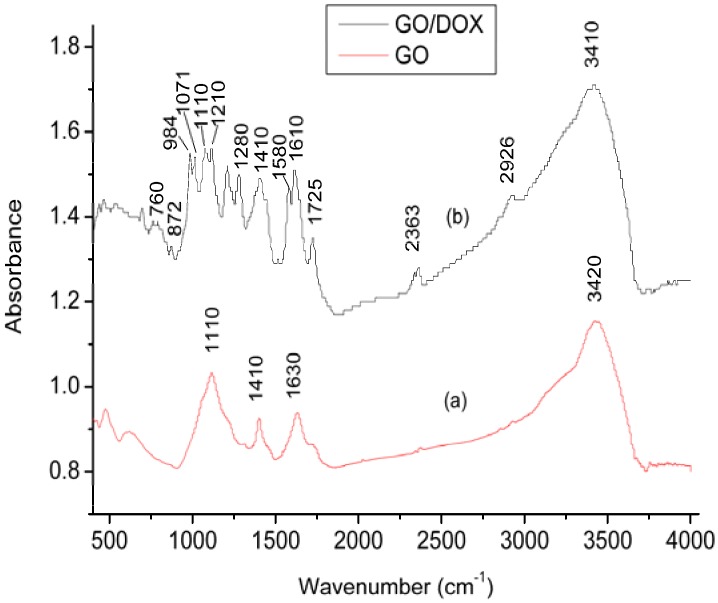
Fourier transform infrared spectroscopy (FTIR) spectra of GO: (**a**) before and (**b**) after doxorubicin hydrochloride (DOX) adsorption.

In order to investigate the functional groups of GO, the Fourier transform infrared spectroscopy (FTIR) spectrum of GO was analyzed and the result is shown in Figure 2a. The broad peak at 3420 cm^−1^ can be attributed to stretching vibration of –OH groups. The peaks at 1630 and 1410 cm^−1^ indicate the existence of asymmetric and symmetric stretching vibration of C=O groups. The peak at 1100 cm^−1^ is assigned to alkoxy C–O groups situated at the edges of the GO sheets [23]. The results suggest that the surface of GO contains oxygen-containing groups such as hydroxyl, carboxylic, epoxy or alkoxy groups introduced by an oxidation process [24]. Figure 2b shows the FTIR spectrum of DOX/GO. The peak of –OH groups has a small shift to the lower band and reaches a value of 3410 cm^−1^. The characteristic peaks at 2926, 1725, 1610, 1410 and 1071 cm^−1^ are assigned to quinone and ketone carbonyl groups [25]. The peak at 2926 cm^−1^ is due to the stretching bands of the C–H groups, The peak at 1725 cm^−1^ is due to the stretching bands of the C=O groups, The peak at 1410 cm^−1^ is due to the stretching bands of the C–C groups, The peak at 1610 cm^−1^ is due to the bending bands of the N–H groups, The peak at 1071 cm^−1^ is due to the stretching bands of the C=O groups. The small peak at 1280 cm^−1^ and 984 cm^−1^ is due to the stretching bands of the C–O–C groups [26], The peaks at 872 and 760 cm^−1^ are due to the primary amine NH_2_ wag and N–H deformation bonds, respectively [27].The additional absorbance bands at the spectrum of DOX/GO confirm the effective loading of DOX on GO.

**Figure 3:**
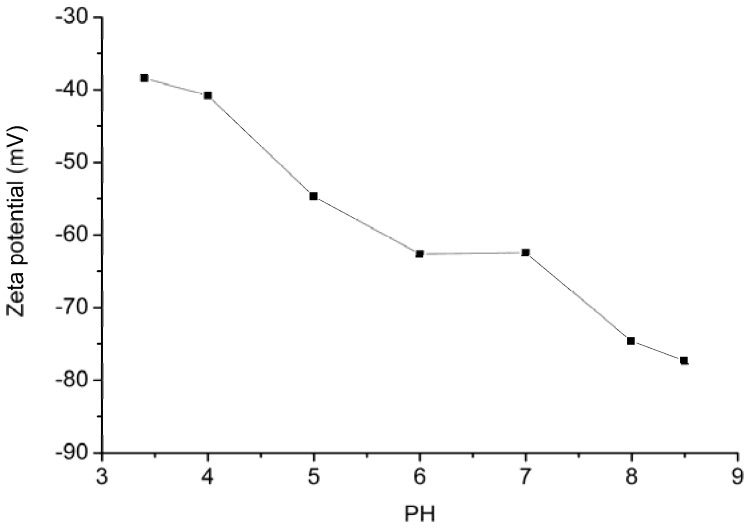
Zeta potential curve *vs.* pH of GO.

Zeta potential can measure the potential difference between the dispersion medium and the stationary layer of fluid attached to the dispersed particle to determine the acidity or basicity of the adsorbent surfaces. The variation of the zeta potentials of GO dependence of pH is shown in Figure 3. It can be seen that GO has negative zeta potentials over the whole tested pH range. It means that the surface charge of GO is negative, which benefits the adsorbtion of cationic molecules [28].

### 2.2. Effect of pH

**Figure 4:**
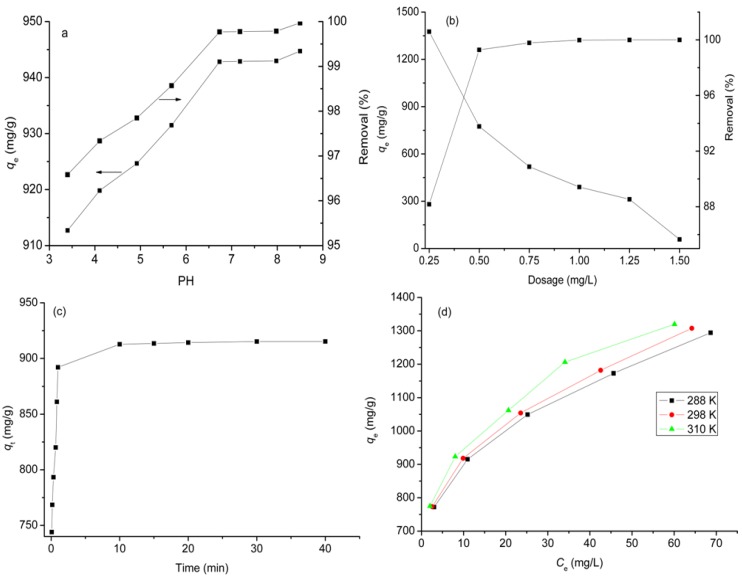
Influences of experimental parameters on the adsorption of DOX on GO: (**a**) pH effect (dosage = 0.5 g/L, temperature = 288 K, concentration = 350 mg/L); (**b**) dosage effect (temperature = 288 K, concentration = 500 mg/L, pH = 3.4); (**c**) contact time on effect (concentration: 350 mg/L; dosage: 0.5 mg/L; temperature: 288 K; pH: 3.4); (**d**) temperature effect (dosage: 0.5 mg/L; temperature: 288 K; pH: 3.4).

The pH of aqueous solution is one of the most important factors in determining the adsorption property of an adsorbent due to its effect on both surface charge of the adsorbent and the degree of ionisation and speciation of the adsorbate. The initial pH effect on DOX adsorbed by GO is shown in Figure 4a. It can be seen that the adsorption capacity is 912.7 mg/g at pH 3.4 and increases with increasing initial pH. At low pH, the increased hydrophilicity and higher solubility of DOX are caused by increased protonation of –NH_2_ groups on DOX, thus leading to the release of more DOX from GO into aqueous solution. The increased adsorption capacity with pH may be due to the change of the zeta potential with variation of pH. At pH 3.4 the zeta potential is −38.4 mV, while it decreases to −77.3 mV at pH 8.5, suggesting that the surface of GO has a more negative charge. The coulombic attraction can readily take place due to the interaction between GO and positively charged DOX molecules [29] at pH 8.5. The percentage removal is very high in the whole pH range and reaches 96.6% at pH 3.4. With increasing pH from 3.4 to 8.5, it has a slight increase and almost reaches 100%. The higher adsorption capacity and percentage removal indicate that GO has high loading efficiency and is a promising drug nanovector. The pH-dependence indicates that acidic conditions benefit the release of DOX from the surface of GO. GO is important in the clinical setting, since the microenvironments of extracellular tissues of tumors and intracellular lysosomes and endosomes are acidic [30].

### 2.3. Effect of Adsorbent Dosage 

The effect of adsorbent dosage on the removal of DOX on GO was studied at pH 3.4 and DOX concentration of 350 mg/L (Figure 4b). On increasing the adsorbent dosage concentration from 0.25 to 0.5 g/L, the percentage removal increased from 88.18% to 99.28%. With further increase in the adsorbent dosage concentration, the percentage removal was not increased significantly. The phenomenon could be explained as a consequence of a partial aggregation of GO at higher concentration, which results in a decrease in effective surface area for the adsorption [31]. The results indicated that the optimum dosage concentration for GO to adsorb DOX for further adsorption experiments is 0.5 g/L.

Figure 4b also shows that the adsorption capacity of GO presents a declining trend with increasing dosage concentration. This trend may be attributed to the decrease in active sites and agglomeration of the adsorbents. On increasing the adsorbent dosage concentration, the active sites of the adsorbent provided are much more than the saturated threshold adsorption points, so only part of the active sites is occupied by DOX molecules, leading to the decrease of adsorption capacity [32].

### 2.4. Effect of Contact Time

The effect of contact time on adsorption of DOX on GO was carried out at DOX concentration of 350 mg/L at 288 K and shown in Figure 4c. It can be seen that the adsorption is very rapid during the first 10 min and the adsorption capacity quickly reaches 912.17 mg/g. The fast adsorption at the initial stage may be due to the special one-atom-thick layered structure of GO, as it becomes in contact with DOX molecules in the aqueous solution, the adsorption occurs immediately due to the higher driving force to make DOX transfer quickly to the active sites on the surface of GO. On further increasing time, the diminishing availability of the remaining active sites and the decrease in the driving force result in a long time to reach equilibrium. Thus, the adsorption rate becomes slower and the adsorption capacity has only a small increase. It only takes 40 min to reach adsorption equilibrium for DOX adsorbed by GO, indicating that GO has high DOX adsorption efficiency.

### 2.5. Effect of Temperature

The temperature of the adsorption medium could be important for energy-dependent mechanisms in the organic molecule adsorption by GO. An increase in the temperature from 288 to 298 K leads to an increase in the adsorption capacity from 1294.05 to 1319.72 mg/g at an equilibrium time of 20 min and initial concentration of 500 mg/L (Figure 4d). The uptake increases with increasing temperature, this effect may be explained by the availability of more inactive sites caused by activation of the adsorbent surface at higher temperatures [33]. The result indicates that the adsorption of DOX on GO is endothermic in nature.

### 2.6. Kinetic Studies 

In order to investigate the mechanism of adsorption and potential rate controlling steps such as chemical reaction, diffusion control and mass transport processes, four kinetic models, namely, pseudo-first-order, pseudo-second-order, Elovich equation, and intra-particle diffusion models were analyzed.

The pseudo-first-order equation is expressed as follows [34]:

(1) $$\log(q_{e}-q_{t})=\log q_{e}-\frac{k_{1}}{2.303}t$$

where *k*_l_ is the Lagergren rate constant of adsorption (1/min). The plot of log (*q*_e_−*q*_t_) against *t* gives a linear relationship from which *k*_1_ and *q*_e_ are determined from the slope and intercept of the plot, respectively.

The pseudo-second-order model can be represented by the following linear form [35]:

(2) $$\frac{t}{q_{t}}=\frac{1}{k_{2}q_{e}^{2}}+\frac{t}{q_{e}}$$

where *k*_2_ is the pseudo second-order rate constant of adsorption (g/mg min). The values of *q*_e_ and *k*_2_ are determined from the slope and intercept of the plot of *t*/*q*_t_ against *t*.

The Elovich model is presented by the following equation [36]:

(3) $$q_{t}=\frac{1}{\beta }\ln\alpha \beta +\frac{1}{\beta }\ln t$$

where *α* is the initial adsorption rate (mg/g·min) and *β* is a desorption constant (g/mg). The plot of *q*_t_ against ln*t* provides a linear relationship in which *α* and *β* are determined from the slope and intercept of the plot.

The intra-particle diffusion model is widely used to predict the rate controlling step [37]. The rate constants of intra-particle diffusion (*k*_i_) at stage *i* are determined using the following equation:

(4) $$q_{t}=k_{i}t^{1/2}+C_{i}$$

where *t*^1/2^ is the square root of the time, *C*_i_ is the intercept at stage *i*. The value of *C*_i_ is related to the thickness of the boundary layer.

**Figure 5:**
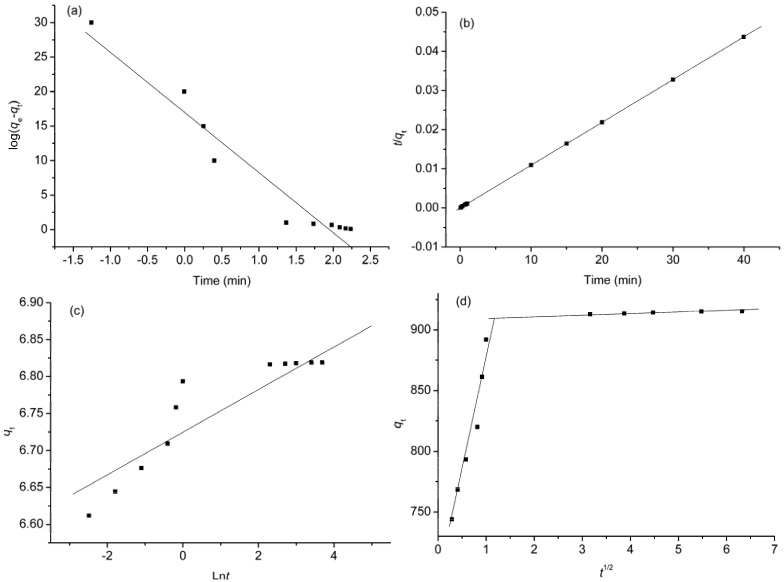
Adsorption kinetics of DOX adsorbed by GO: (**a**) pseudo-first-order; (**b**) pseudo-second-order; (**c**) Elovich; and (**d**) intraparticle diffusion models.

The validity of each kinetic model was checked by the fitted straight lines and is depicted in Figure 5a–d. The corresponding kinetic parameters and the determination coefficients are summarized in Table 1. The values of the determination coefficient obtained from the linear plot of pseudo-first-order (Figure 5a) and Elovich (Figure 5c) models are very small (*R*^2^ = 0.9467 and 0.7801), suggesting that the applicability of these two models to the adsorption processes of DOX on GO is unfeasible. The higher determination coefficient (*R*^2^ = 1.0000) is obtained for pseudo-second-order model (Figure 5b), indicating that the experimental data are well described by pseudo-second order model and the nature of adsorption is a chemical-controlling process [38].

**Table 1:** Parameters of four kinds of kinetic models.

Kinetic model	Parameters	Values
Pseudo-first-order	*q*_e_ (mg/g) × 10^16^	7.6 × 10^16^
	*k*_1_ (1/min)	19.56
	*R*^2^	0.9467
Pseudo-second-order	*q*_e_ (mg/g)	909.09
	*K*_2_ (g/mg min)	0.03
	*R*^2^	1.0000
Elovich	Ln*α*	229.11
	*Β*	34.60
	*R*^2^	0.7801
Intra-particle diffusion	*k*_I_	190.85
	*C*_I_	685.82
	*R*^2^	0.9529
	*k*_II_	0.84
	*C*_II_	910.28
	*R*^2^	0.9353

The intra-particle diffusion model can be reasonably utilized to elaborate the adsorption mechanism. Figure 5d shows that the plots of *q*_t_ *vs. t*^1/2^ show multi-linearity characterizations, indicating that more than one diffusion step takes place. The first section is sharper and does not pass through the origin. It indicates that intra-particle diffusion is not only the rate-controlling step, but is also affected by the boundary layer diffusion process [37]. The second subdued portion is the gradual adsorption stage, where intra-particle diffusion starts to slow down due to the extremely low DOX concentration left in solution.

### 2.7. Adsorption Isotherms

Several mathematical models have been used for describing equilibrium studies for the adsorption of drugs onto solid surfaces, and the Freundlich and Langmuir models are frequently utilized to fit the experimental data. In this work, both models were applied to describe the experimental data obtained at 288, 298 and 310 K.

Freundlich expression is an empirical equation based on adsorption on a heterogeneous surface as well as multilayer sorption. The equation is commonly expressed as follows [39]:

(5) $$\text{Ln}q_{\text{e}}=\mathrm{Ln}k_{\text{F}}+\frac{1}{n}\mathrm{Ln}C_{e}$$

where *k*_F_ is a Freundlich constant related to adsorption capacity (L/g), 1*/n* is an empirical parameter related to adsorption intensity. The Freundlich constants can be calculated from the plots of Ln*q*_e_ *vs.* Ln*C*_e_. 

The Langmuir model is the simplest theoretical model for monolayer adsorption on a surface with a finite number of identical sites. It assumes that the adsorption occurs on a homogenous surface and there is no interaction between adsorbates in the plane of the surface. The equation of the Langmuir isotherm is as follows [40]:

(6) $$\frac{C_{e}}{q_{e}}=\frac{C_{e}}{q_{\max}}+\frac{1}{q_{\max}k_{L}}$$

where *C*_e_ is the equilibrium concentration of the solution (mg/L), *q*_max_ is the maximum adsorption capacity (mg/g), *k*_L_ is a Langmuir constant related to the affinity of the binding sites and energy of adsorption (L/g). The Langmuir constants can be obtained from the plot of *C*_e_/*q*_e_ *vs. C*_e_.

The isotherm constants and determination coefficients are summarized in Table 2. The adsorption data of DOX on GO fit well with the Langmuir isotherm model with high *R*^2^ (0.9924–0.9941). This result may be due to the homogeneous distribution of active sites on the edge and two sides of the GO sheet. Table 2 also shows that GO has the maximum DOX adsorption capacity of 1428.57 mg/g. It is apparent that the DOX loading on GO can reach 142.86 wt %, which is higher than those of the commonly used drug carriers such as polymersomes (47 wt %) [41], hydrogel microspheres (70 wt %) [42], and BSA–dextran–chitosan nanoparticles (56 wt %) [43]. It suggests that GO is a promising drug carrier and has great potential application in preparation of drug delivery systems.

**Table 2:** Isotherm parameters for the adsorption of doxorubicin hydrochloride (DOX) on graphene oxide (GO).

Temperature (K)	Langmuir			Freundlich		
	*q*_max_	*k*_L_	*R*^2^	1/*n*	*k*_F_	*R*^2^
288	1428.57	0.20	0.9930	0.16	632.96	0.9884
298	1428.57	0.22	0.9924	0.16	651.06	0.9851
310	1428.57	0.26	0.9941	0.16	678.78	0.9866

### 2.8. Thermodynamic Study

**Figure 6:**
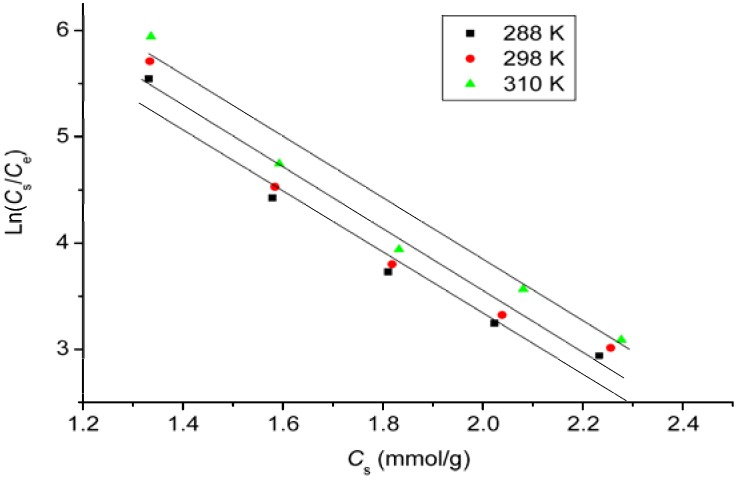
Plots of ln*q*_e_/*C*_e_ *vs.* 1/*T* for DOX adsorbed by GO.

In the adsorption reaction, thermodynamic parameters are used to judge whether the reaction occurs spontaneously or not. Thermodynamic parameters can be calculated from the variation of the thermodynamic equilibrium constant *K*_0_ with the change in temperature [21]. For adsorption reactions, *K*_0_ is defined as follows:

(7) $$K_{0}=\frac{a_{s}}{a_{e}}=\frac{v_{s}}{v_{e}}\frac{C_{s}}{C_{e}}$$

where *a*_s_ is the activity of adsorbed DOX, *a*_e_ is the activity of DOX in solution at equilibrium, *C*_s_ is the amount of DOX adsorbed by per mass of GO (mmol/g), *v*_s_ is the activity coefficient of the adsorbed DOX and *v*_e_ is the activity coefficient of DOX in solution. As DOX concentration in the solution decreases and approaches zero, *K*_0_ can be obtained by plotting ln(*C*_s_/*C*_e_) *vs. C*_s_ and extrapolating *C*_s_ to zero (Figure 6).

The average standard enthalpy change ($\Delta H^{0}$) is obtained from the Van’t Hoff equation:

(8) $$\ln K_{0}(T_{3})-\ln K_{0}(T_{1})=\frac{-\Delta H^{0}}{R}(\frac{1}{T_{3}}-\frac{1}{T_{1}})$$

where *T*_3_ and *T*_1_ are two different temperatures, and *R* is the universal gas constant (8.314 J/K·mol).

The adsorption standard free energy changes ($\Delta G^{0}$) can be calculated according to:

(9) $$\Delta G^{0}=-RT\ln K_{0}$$

The standard entropy change ($\Delta S^{0}$) can be obtained by:

(10) $$\Delta S^{0}=-\frac{\Delta G^{0}-\Delta H^{0}}{T}$$

The thermodynamic parameters were calculated and are listed in Table 3. The negative values of $\Delta G^{0}$ at three tested temperatures reveal that the adsorption process is spontaneous. The positive values of $\Delta H^{0}$ suggest that the interaction of DOX adsorbed by GO is an endothermic process, the positive values of $\Delta S^{0}$ indicate increased randomness at the adsorbent/solution interface during the adsorption of DOX on GO [21].

**Table 3:** Thermodynamic parameters for DOX on GO.

Thermodynamic constant	Temperature (K)		
	288	298	310
*K*_0_	9256	10952	14657
Δ*G*^0^ (kJ/mol)	−21.87	−23.04	−24.72
Δ*H*^0^ (kJ/mol)	15.52	15.52	15.52
Δ*S*^0^ (J/mol·K)	129.83	129.36	129.81

### 2.9. Adsorption Mechanisms

The possible mechanisms of high adsorption capacity and fast adsorption rate of DOX on GO may be elucidated by the following explanations:

(a) The unique structure of GO. GO is a one-atom-thick two-dimensional individual sheet. Every exposed carbon atom has the opportunity to contact and interact with DOX molecules. The adsorption isotherm data, fitted well with the Langmuir equation (Table 2) indicates that a monolayer of DOX molecules is adsorbed homogeneously on the edge and two sides of a GO sheet.
(b) Electrostatic interaction. GO has a negatively charged surface. The positively charged DOX molecules can be easily attracted and adsorbed onto the surface of GO by means of electrostatic attraction [44].
(c) (c) The role of hydrogen bonding. The edge of GO has many oxygen-containing functional groups such as −COOH, −C=O and −OH. The functional groups make GO more hydrophilic and suitable for the adsorption of relatively low molecular weight compounds [45] through the role of hydrogen bonding. The hydrogen bonding between DOX and GO maybe exists in four formats: (i) –COOH of GO and –OH of DOX; (ii) –COOH of GO and –NH_2_ of DOX; (iii) –OH of GO and –OH of DOX; and (iv) –OH of GO and –NH_2_ of DOX [25].

### 2.10. Desorption Studies

**Figure 7:**
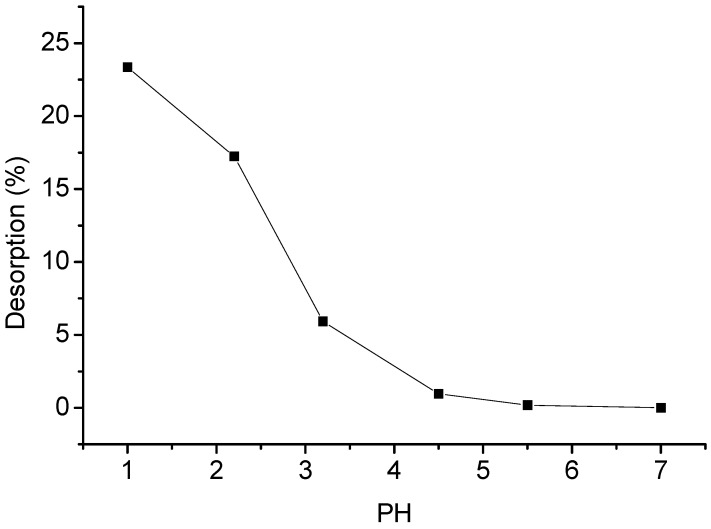
Desorption of DOX from GO by adjusting the pH values of the solution using HNO_3_ and NaOH solutions.

Figure 7 shows the DOX desorption curve variation on changing the solution pH values. It is apparent that DOX desorption increases with increasing pH value. The desorption percentage increases from 0 to 23% as the solution pH decreases from 7.0 to 1.0. The result shows that DOX adsorbed by GO can partially be desorbed at acidic conditions (close to gastric juice 0.9–1.5), suggesting that GO is a promising drug carrier and has great potential application in preparation of drug delivery systems.

## 3. Experimental Section 

### 3.1. Materials

Expandable graphite was purchased from Henglide Graphite Co., Ltd, Qingdao, China. Concentrated sulfuric acid (H_2_SO_4_), hydrogen peroxide (H_2_O_2_), potassium permanganate (KMnO_4_), sodium nitrate (NaNO_3_), hydrochloric acid (HCl) were of analytical grade and purchased from Sinopharm Chemical Reagent Co., Ltd, China. DOX was obtained from Zhejiang Hisun Pharmaceutical Co., Ltd., Taizhou, China.

GO was synthesized by oxidation of expandable graphite according to a modified Hummers method [46]. In brief, expandable graphite (5 g) was mixed with a mixture of 230 mL H_2_SO_4_, KMnO_4_ (30 g) and NaNO_3_ (5 g) in an ice bath. The ice bath was then removed and the obtained mixture was kept at 273 K for 24 h. Later on the mixture was stirred at 308 K for 30 min and then slowly diluted with deionized water. The reaction temperature was rapidly increased to 371 K and kept for 15 min. Then 30% H_2_O_2_ was added to the mixture causing the color to turn yellow along with bubbling. Finally, the mixture was centrifuged and washed with HCl (5%) and deionized water several times to obtain GO.

### 3.2. Adsorbent Characterization

TEM (JEM-2100F) was employed to examine the structure and morphology of GO. Bulk dry weight-based C, H, and N contents of graphene were determined using an elemental analyzer (Elementar, Germany) with the oxygen content calculated by mass difference. The Brunauer–Emmett–Teller (BET) surface area, pore volume, and pore diameter of GO were determined from N_2_ adsorption at −77 K using a Micrometric ASAP 2000 system. Functional groups of GO and DOX adsorbed by GO (DOX/GO) were analyzed by a Perkin-Elmer-283B FTIR spectrometer within the wavenumber range 400–4000 cm^−1^. The values of zeta potential were measured by a Malvern zetameter (Zetasizer 2000). They are determined byh adjusting the solution (10 mM NaCl background electrolyte) pH from 3.4 to 8.5 by adding 0.1 M nitric acid or sodium hydroxide solution to the glass beaker at 288 K.

### 3.3. Adsorption and Desorption Experiments

A stock solution of DOX (1000 mg/L) was prepared and further diluted to the desired concentrations before use. The batch adsorption experiments were conducted by adding 5 mg GO into 10 mL solutions with DOX concentrations from 300 to 500 mg/L. The solutions were shaken on a temperature-controlled water bath shaker (SHZ-82A). All experiments were performed at room temperature (288 K) except for thermodymic studies and the solution pHs were not adjusted (3.4) except for studying pH effect. The initial pH of the solution was 3.4 after adsorption equilibrium, the solutions were centrifugated at 10,000 rpm for 6 min and final DOX concentrations of supernatants were determined by measuring the absorbance of the samples at wavelength of 478 nm by a UV–Vis spectrophotometer (TU-1810, Beijing Purkinje General Instrument Co., Ltd., Beijing, China). The adsorbed amount of DOX on GO was calculated according to the following equation:

(11) $$q_{e}=(\frac{C_{0}-C_{e}}{m})V$$

where *C*_0_ and *C*_e_ are the initial and equilibrium concentrations of DOX in solutions (mg/L), *V* is the volume of solution (L), *m* is the mass of adsorbent (g).

The effect of pH on DOX adsorbed by GO was studied in an initial pH range of 3.4–8.5 using an Orion 4-Star pH meter. The pH of 10 mL solution with DOX concentration 350 mg/L was adjusted using appropriate concentrations of HNO_3_ or NaOH. In dosage studies, different amounts (2.5–15 mg) of adsorbents were added into 10 mL solutions with DOX concentration of 350 mg/L.

The effect of contact time on the adsorption of DOX was studied by adding 5 mg of GO into 10 mL DOX solutions (350 mg/L). At predetermined time intervals, the samples of these solutions were withdrawn from the shaker and the DOX solutions were separated by centrifugation at 10,000 rpm for 6 min and measured. The amounts of DOX adsorbed by GO were calculated using the following equation:

(12) $$q_{t}=\frac{\left(C_{0}-C_{t}\right)}{m}V$$

where *C*_0_ and *C*_t_ are the initial and the predetermined time concentrations of DOX in solutions (mg/L), *V* is the volume of solution (L), *m* is the mass of adsorbent (g).

To evaluate the thermodynamic properties, 5 mg adsorbents were added into 10 mL solutions with initial DOX concentrations ranging from 300 to 500 mg/L. These samples were shaken to reach equilibrium at 288, 298 and 310 K, respectively.

In order to investigate the desorption properties of DOX from GO, 5 mg GO was added into 10 mL solution with initial DOX concentration of 350 mg/L at pH 3.4. As the adsorption reached equilibrium, the mixture was centrifuged to remove free DOX in the supernatant and then redispersed in 10 mL deionized water. The pH values of the solutions were adjusted, from 1.0 to 7.0, using HNO_3_ and NaOH solutions, respectively. After 2 h, the DOX concentrations were remeasured and the desorption results were then obtained.

## 4. Conclusions

The ability of GO to adsorb DOX was studied in a batch system. The experimental results showed that the adsorption of DOX on GO was dependent on adsorbent dosage, contact time, pH and temperature. The equilibrium data followed the Langmuir isotherm model better than the Freundlich model. The kinetic studies showed that GO had rapid adsorption rate and efficiency and the kinetic data fitted well to a pseudo-second-order model. The thermodynamic parameters indicated that the adsorption of DOX on GO was an endothermic and spontaneous process. The possible adsorption mechanisms of DOX on GO maybe due to the unique one-atom-thick structure, electrostatic interaction, hydrogen bonding and π–π stacking interaction. The studies provide fundamental understanding of the adsorption mechanism between DOX and GO and benefit the development of GO based drug delivery systems.
